# Effect of Organic or Inorganic Mineral Premix in the Diet on Laying Performance of Aged Laying Hens and Eggshell Quality

**DOI:** 10.3390/ani12182378

**Published:** 2022-09-12

**Authors:** Chan-Ho Kim, So Hee Jeong, Se Jin Lim, Si Nae Cheon, Kihyun Kim, Julan Chun, Junghwan Jeon

**Affiliations:** Animal Welfare Research Team, National Institute of Animal Science, Rural Development Administration, Wanju 55365, Korea

**Keywords:** inorganic mineral, organic mineral, broken-shell less egg, eggshell strength, laying hens

## Abstract

**Simple Summary:**

Organic trace minerals have a greater impact on eggshell quality than inorganic trace minerals. Trace minerals involved in animal growth and development, including eggshell formation, are essential for laying hens. However, most previous research center around the effects of single trace minerals or single aspects of mineral supplementation. The effect of synchronous replacement of inorganic minerals, such as iron, copper, zinc, manganese, and magnesium, with organic trace minerals on the quality of eggs laid by aged laying hens, has not been evaluated extensively. This study concludes that feed with organic trace minerals improved eggshell quality of aged laying hens.

**Abstract:**

In this study, we examined the effect of diets supplemented with organic and inorganic mineral premixes on the laying performance and eggshell quality of aged laying hens. A total of 600 68-week-old Hy-Line Brown laying hens were randomly assigned to 1 of 3 dietary treatments, repeated 5 times: Mash type basal diet, basal diet supplemented with an inorganic mineral premix (1.0 g/kg), and basal diet supplemented with an organic mineral premix (1.8 g/kg). The results showed that eggshell strength was higher (*p* < 0.01) in the inorganic mineral diet group than in the organic mineral and basal diet groups. Further, the levels of Fe and Mn in the liver were higher (*p* < 0.05) in the inorganic and organic mineral diet groups than in the basal diet group. The concentrations of Fe and Mg in the spleen were different (*p* < 0.05) among the treatment groups, with the highest levels reported in the organic mineral premix group. The concentrations of Cu, Zn, and Mn in the eggshell were different (*p* < 0.05) among the groups, with the highest levels reported in the inorganic and organic mineral premix diet groups. In conclusion, a diet containing organic mineral premix improved eggshell strength and had no detrimental effect on the laying performance of aged laying hens.

## 1. Introduction

Egg producers are constantly looking for ways to improve their economic benefits by improving the production and quality of their eggs and maintaining the health of their hens. A total of 8% of all egg losses are due to poor egg quality [1]. Eggshell quality is essential for eggs to effectively defend against invading pathogens such as *Salmonella* sp. In addition, as hens grow older, their eggshell size increases, but their weight does not increase. This indicates a decrease in eggshell strength [2]. One of the primary concerns in egg production is the decrease in eggshell quality due to increased hen age, poor environmental conditions, and insufficient minerals in the diet [3]. In laying hens, trace minerals are directly involved in eggshell and bone formation [4]. Trace minerals directly interact with calcium crystals during eggshell formation and affect eggshell quality [5,6]. Currently, the forms of minerals added to livestock feed are inorganic forms such as oxides, sulfates, carbonates and phosphates. [1]. The antagonism of inorganic minerals can reduce absorption, requiring commercial feeds to be supplied with excess inorganic minerals to prevent deficiencies, which is an environmental pollution concern [7]. The usage of higher than recommended amounts of minerals has been criticized for the resulting environmental implications linked with their accumulation in soil [8]. Chelated or complex trace elements may improve the bioavailability of minerals for pigs and poultry [9,10,11]. Some studies have reported that organic mineral material has an improvement effect on eggshell quality compared to inorganic mineral material [5]; however, most mineral research has focused on single-supplementation effects. In this study, we evaluated the effect of diets supplemented with multiple organic and inorganic trace minerals, including iron, copper, zinc, manganese, and magnesium, on the quality of eggs of aged laying hens.

## 2. Materials and Methods

The animal experiment in this study was conducted with the approval of the Chung-Ang University Animal Experiment Committee (IACUC No. 201100101).

### 2.1. Preparation of Mineral Proteinate

Trace mineral (iron, copper, zinc, manganese, and magnesium) soy proteinate was developed in the animal nutrition laboratory at Chung-Ang University [12]. Soybean meal digest was prepared by hydrolysis of soybean meal mixed with an enzyme (Alkalase^®^ 2.4 L; Novozymes, Bagsvaerd, Denmark) under an aqueous condition of pH 8 and at a temperature of 60 °C for 8 h. Then, FeSO4·7H_2_O, CuSO_4_·H_2_O, ZnSO_4_·H_2_O, MnSO_4_·H_2_O, and MgSO_4_·7H_2_O were allowed to react with the soybean meal digest at a 1:1 weight ratio at pH values of 8.8, 8.4, 8.8, 8.3, and 8.7, respectively. The precipitates were separated and dried. The final products contained 191 g/kg iron, 174 g/kg copper, 180 g/kg zinc, 168 g/kg manganese, and 90 g/kg magnesium, respectively.

### 2.2. Bird and Experimental Design

A total of 600 68-week-old Hy-Line Brown laying hens were randomly assigned to 1 of 3 dietary treatments. Each treatment consisted of 5 replications of 20 cages of two hens per cage (30 cm × 37 cm × 40 cm = width × length × height). The basal diet was a commercial-type formulated to exceed the nutrient recommendations of the NRC [13] for laying hens, with iron, copper, zinc, manganese, and magnesium concentrations of 93.3 mg/kg, 13.2 mg/kg, 24.6 mg/kg, 22.8 mg/kg, and 2113 mg/kg, respectively, (Table 1). The other two diets were prepared by adding an inorganic mineral premix (sulfate and oxide) or the organic mineral premix (soy-proteinate) to the basal diet. The analyzed concentrations of iron, copper, zinc, manganese, and magnesium in the inorganic and organic diets were 92.2 or 93.3 mg/kg, 13.0 or 13.2 mg/kg, 24.6 or 24.8 mg/kg, 23.7 mg/kg, and 2125 mg/kg, respectively. During the experimental period of 5 weeks, feed and water ad libitum and the light were exposed to a 16L:8D schedule. Temperature and humidity in the laying house were maintained at 21 ± 3 °C and 60–70%, respectively.

### 2.3. Chemical Analysis and Data Collection

To assess egg productivity, hen-day productions, mean egg weight, and broken and shell-less egg productions were recorded daily and used to calculate a weekly average. The feed intake was measured weekly, and the feed conversion ratio (feed intake/100 g egg mass) was calculated. The data collected from the layers that died during the experiment were excluded. Ten eggs per replicate were randomly collected every week to measure individual weight followed by examination of the external (eggshell strength, thickness, and color) and internal (egg yolk and Haugh unit) quality. Eggshell strength was measured using the Texture Systems Compression Test Cell (Model T2100C; Food Technology Co., Ltd., Rockville, MD, USA). Eggshell thickness (without the inner and outer shell membranes) was determined as the mean of the values recorded at three locations on the eggshell (air cell, equator, and sharp end) using a dial pipe gauge (Model 7360; Mitutoyo Co., Ltd., Kawasaki, Japan); the following formula was used for this purpose [14]: Eggshell thickness = (sharp point thickness + equator point thickness + air cell thickness)/3. The Hunter Lab colors of the eggshells were measured using a Chroma meter (Model CR-400, Minolta, Osaka, Japan). Egg yolk color was evaluated using the Roche color fan (Hoffman- La Roche Ltd., Basel, Switzerland), where 1 = light pale and 15 = dark orange. To determine the Haugh unit, each egg was weighed and broken carefully on a flat surface. The albumen height was measured using a micrometer (Model S-8400; Ames, Waltham, MA, USA), and the Haugh unit was determined using the following equation: HU = 100 log_10_ (H-1.7W^0.37^ + 7.56), where H is the observed height of albumen in millimeters and W is the weight of the egg in grams [15].

### 2.4. Collection of Spleen, Liver, Eggshell, and Blood Samples

At the end of the experiment period, the blood samples were collected, using EDTA-treated BD Vacutainer^®^ tubes (Becton Dickinson, Franklin Lakes, NJ, USA), from the wing vein of 10 birds randomly selected from each treatment. The tubes were placed on ice, and the whole-blood samples were immediately evaluated. The white and red blood cell, heterophil, and lymphocyte counts and hemoglobin and hematocrit levels were analyzed using the Hemavet Multispecies Hematology System (Drew Scientific Inc., Oxford, CT, USA). The H/L ratio was calculated by dividing lymphocytes in heterophils. 

At the end of the experiment, 10 birds per treatment were sacrificed by cervical dislocation for spleen and liver sampling. The spleens and livers were washed extensively under running water and immediately stored in individual sample bags at −20 °C. The samples were dried for 4 days at 50 °C. The dried samples were cooled to room temperature (20 °C), pulverized, bagged, and stored in a refrigerator until chemical analysis of iron, copper, zinc, manganese, and magnesium levels. After the end of the experiment, a total of 60 eggs were randomly selected for each treatment group, 20 each, and the shell was separated from the membrane. The eggshells were dried at 105 °C for 1 day. The concentrations of iron, copper, zinc, manganese, and magnesium in the spleen, liver, eggshell, and whole blood were determined by ICP spectrometry (Optima 5300 DV) after wet-ash digestion with nitric/perchloric acid [16]. 

### 2.5. Statistical Analysis

All data were analyzed by analysis of variance (ANOVA) with a fully randomized design using the Proc Mixed procedure from SAS (SAS Inst., Inc., Cary, NC). Outlier data were identified using the UNIVARIATE procedure in SAS and no outliers were found. Differences among the least squares means were evaluated using the PDIFF option with Tukey’s adjustment. In this process, the output values were converted to letter groups using a macro program [17]. The LSMEANS procedure was used to calculate the means of either basal diet vs. mineral premix or inorganic mineral premix vs. organic mineral premix. The significance and trend of statistical tests were set at *p*-values < 0.05 and 0.05 ≤ *p* ≤ 0.10, respectively.

## 3. Results

### 3.1. Laying Performance and Eggshell Quality

During the 5 weeks of the experiments, the hen-day egg production rates, egg weights, average daily feed intake, and feed conversion ratios between the inorganic and organic mineral diet groups were not affected (Table 2). However, the broken and shell-less egg rates were lower (*p* < 0.01) in the inorganic and organic mineral premix groups than in the basal diet group. Eggshell strength was higher (*p* < 0.01) in the inorganic mineral premix group than in the organic mineral premix and basal diet groups. Eggshell thickness, eggshell color (Hunter L*, a*, and b*), egg yolk color, and Haugh units were not significantly influenced by the inorganic and organic mineral diets (Table 3).

### 3.2. Hematological Analysis

There were no significant differences in the white and red blood cell, heterophil, and lymphocyte counts, H/L ratios, and hemoglobin and hematocrit values among the groups (Table 4).

### 3.3. Mineral Concentrations in Spleen, Liver, Eggshell, and Whole Blood

We found that the concentrations of iron and manganese were higher in the inorganic diet group (*p* < 0.05) than in the organic mineral premix and basal diet groups, but no differences were observed in the concentrations of copper, zinc, and magnesium. The concentrations of iron and magnesium in the spleen were different (*p* < 0.05) among the treatment groups, with the highest values reported in the organic mineral premix group. The concentrations of copper, zinc, and manganese in the eggshell differed (*p* < 0.05) among the groups, with the highest values reported in the inorganic and organic mineral premix groups (Table 5). No differences were observed in the blood concentrations of iron, copper, zinc, manganese, and magnesium among the treatment groups. 

## 4. Discussion

### 4.1. Laying Performance and Eggshell Quality

Dietary iron, copper, zinc, manganese, and magnesium concentrations are related to laying performance and eggshell quality [18,19,20,21,22]. Similar improvements in eggshell strength and broken and shell-less egg rates were observed among the three groups in the current experiment, which may be attributed to the high magnesium content in the eggshell [21,22]. Zamani et al. [23] reported that a diet with a manganese concentration of 30 mg/kg improved eggshell quality. Therefore, manganese may be the mineral responsible for improving eggshell strength. Similarly, broken egg and shell-less egg production rates decreased in the mineral premix diet group, possibly due to improved eggshell strength. Some research has reported that organic trace elements in the nutrition of laying hens have a positive impact on egg quality. Gheisari et al. [24] found that the inclusion of organic zinc, manganese, and copper in a corn-soybean diet improved eggshell quality. In addition, Favero et al. [25] reported that when zinc, manganese and copper were fed to broiler breeders in the form of organic amino acids, eggshell quality improved and early embryonic mortality was reduced. Zinc, copper, and manganese from organic (amino-acid-metals chelates) or inorganic sources (ZnSO_4_·H_2_O, CuSO_4_·5H_2_O, and MnO) influence the mechanical properties of eggshells by affecting calcite crystal formation and the crystallographic structure of the eggshells [18]. In addition, chelated minerals are transported and absorbed by amino acids. Therefore, since it uses the same absorption pathway as the amino acid to which it is bound, it has the advantage of increased absorption than the inorganic form. This reduces competition for inorganic transmineral binding sites and ultimately reduces excretion of minerals through bile and feces [1,26]. According to some reports, organic trace minerals are more bioavailable than inorganic forms [27,28], whereas others have not found any difference between the two forms [29,30]. In our study, inorganic and organic mineral supplementation had no difference on the laying performance (hen-day egg production, egg weight, feed intake, FCR, and eggshell color). This result is consistent with those of previous studies using hens of various ages and diets supplemented with levels of the inorganic and organic minerals (iron, copper, zinc, manganese, and magnesium) exceeding the minimum requirements [19,20].

### 4.2. Hematological Analysis 

Blood parameters are excellent indicators of average body condition, reflecting physiological, nutritional, and pathological changes [31]. The leukocyte count has been used as a measure of immune function in birds [32]. Hosienpour et al. [33] reported that the supplementation of organic minerals did not affect the hematological parameters of lamb. In contrast, previous research reported that supplementation of organic trace minerals increased RBV parameters and hematological response in animals [34,35]. However, further studies are needed to determine the effects of various forms of mineral premix in the diet on poultry hematological parameters.

### 4.3. Mineral Concentrations in Spleen, Liver, Eggshell, and Blood Samples

The concentration of mineral (copper, zinc, manganese and magnesium) content of eggshell was significantly increased by 3.16%, 5.22%, 4.31% and 5.26%, respectively, in organic form compared with inorganic form. These results are consistent with those of Idowu et al. [36], who reported that supplementation of the diet of laying hens with organic zinc (140 mg/kg Zn proteinate) increased zinc accumulation in the eggshell compared to controls and supplementation with inorganic sources (ZnCl_2_, ZnSO_4_, ZnO, and ZnCO_3_). In another previous study, in contrast with our results, supplementation with organic copper led to an increase in the copper concentration in the eggshell compared with supplementation with inorganic copper [37]. Therefore, blood samples may be a better indicator of trace-element bioavailability than eggshells and may have produced different results, despite the tendency for varying blood concentrations of trace elements in [38]. Ao et al. [39] reported that diets with a high level of organic minerals decreased the concentration of copper in the liver owing to the sequestration of dietary copper by intestinal metallothionein. This indicates that increased zinc absorption leads to increased intestinal metallothionein formation.

## 5. Conclusions

In the current study, the supply of iron, copper, zinc, manganese and magnesium in the organic form as feed sources was superior to the inorganic form of minerals, and the supply of organic minerals in the form of organic matter can be used without adversely affecting egg productivity and egg quality. Therefore, eggshell strength can be improved by supplying minerals in an organic form.

## Figures and Tables

**Table 1:** Composition and nutrient content of experimental diets.

	Basal	Inorganic	Organic
Ingredient (g/kg)			
Corn	412.5	411.5	411.1
Wheat	150.0	150.0	150.0
Soybean meal	250.0	250.0	250.0
Corn distillers dried grains with soluble	50.0	50.0	50.0
Canola meal	20.0	20.0	20.0
Tallow	5.0	5.0	5.0
Molasses	5.0	5.0	5.0
Limestone	97.0	97.0	97.0
Dicalcium phosphate	7.0	7.0	7.0
Sodium chloride	2.0	2.0	2.0
Vitamin premix ^1^	1.5	1.5	1.5
Inorganic mineral premix ^2^	-	1.0	-
Organic mineral premix ^3^	-	-	1.8
Total	1000.0	1000.0	1000.0
Calculated composition ^4^			
ME, MJ/kg	11.32	11.32	11.32
Crude protein, g/kg	186.0	186.0	186.0
Calcium, g/kg	38.0	38.0	38.0
Available phosphate, g/kg	3.3	3.3	3.3
Lysine, g/kg	9.7	9.7	9.7
Methionine, g/kg	3.1	3.1	3.1
Iron, mg/kg	93.3	93.7	93.7
Copper, mg/kg	13.2	13.3	13.3
Zinc, mg/kg	24.6	25.2	25.2
Manganese, mg/kg	22.8	23.7	23.7
Magnesium, g/kg	2113	2125	2125
Analyzed composition ^5^			
Iron, mg/kg	92.3	92.2	93.1
Copper, mg/kg	12.9	13.0	13.2
Zinc, mg/kg	24.2	24.6	24.8
Manganese, mg/kg	22.5	22.9	23.2
Magnesium, g/kg	2115	2123	2120

^1^ Provided per kilogram of the complete diet: vitamin A (from vitamin A acetate), 12,500 IU; vitamin D_3_, 2500 IU; vitamin E (from DL-α-tocopheryl acetate), 20 IU; vitamin K_3_, 2 mg; vitamin B_1_, 2 mg; vitamin B_2_, 5 mg; vitamin B_6_, 3 mg; vitamin B_12_, 18 µg; calcium pantothenate, 8 mg; folic acid, 1 mg; biotin, 50 µg; niacin, 24 mg. ^2^ Provided per kilogram of complete diet: Fe (as FeSO_4_·7H_2_O), 40 mg; Cu (as CuSO_4_·H_2_O), 8 mg; Zn (as ZnSO_4_·H_2_O), 60 mg; Mn (as MnSO_4_·H_2_O) 90 mg; Mg (MgO) as 1500 mg. ^3^ Provided per kilogram of complete diet: Fe (as Fe-soy proitenate), 40 mg; Cu (as Cu-soy proteinate), 8 mg; Zn as Zn-soy proteinate), 60 mg; Mn (as Mn-soy proteinate), 90 mg; Mg as (Mg-soy proteinate), 1500 mg. ^4^ Nutrient contents in all diet were calculated. ^5^ Analyzed in triplicated.

**Table 2:** Effect of dietary supplementation with different mineral premixes on laying performance ^1^.

Items	Dietary Treatment ^2^			SEM ^3^	*p*-Value	
	Basal	Inorganic	Organic		Basal vs. Mineral	Inorganic vs. Organic
Hen-day egg production, %	83.2	85.4	83.9	0.835	0.19	0.22
Egg weight, g	65.4	65.9	65.1	0.419	0.84	0.21
Feed intake, g	124.4	126.5	124.6	1.723	0.42	0.25
Feed conversion ratio, g/g	2.29	2.25	2.28	0.036	0.66	0.49
Broken and shell-less eggs, %	0.55 ^a^	0.15 ^b^	0.14 ^b^	0.068	<0.01	0.95

^a,b^ Means in the same row with different superscripts differ significantly (*p* < 0.05). *n* = 5. ^1^ Data are least squares means of 5 replicate per treatment. ^2^ Basal = basal diet (no inclusion mineral premix); Inorganic = 1.0 g/kg inorganic mineral premix; Organic = 1.8 g/kg organic mineral premix. ^3^ Standard error of means.

**Table 3:** Effects of dietary supplementation with different mineral premixes on egg quality ^1^.

Items	Dietary Treatment ^2^			SEM^3^	*p*-Value	
	Basal	Inorganic	Organic		Basal vs. Mineral	Inorganic vs. Organic
Eggshell strength, kg/cm^2^	3.40 ^b^	3.54 ^ab^	3.69 ^a^	0.045	<0.01	0.12
Eggshell thickness, μm	358.7	361.9	361.1	3.288	0.63	0.61
Eggshell color						
CIE L*	52.9	54.8	54.5	0.799	0.10	0.79
CIE a*	13.8	14.0	14.6	0.361	0.24	0.24
CIE b*	20.1	20.3	20.4	0.149	0.20	0.62
Egg yolk color	10.2	10.1	10.1	0.131	0.67	0.99
Haugh unit	81.9	82.5	83.7	0.839	0.29	0.32

^a,b^ Values in the same row with different superscripts differ significantly (*p* < 0.05). *n* = 30. ^1^ Data are presented as least squares means of 30 replicates per treatment. ^2^ Basal = basal diet (no mineral premix added); inorganic = 1.0 g/kg inorganic mineral premix; organic = 1.8 g/kg organic mineral premix. ^3^ Standard error of means.

**Table 4:** Effects of dietary supplementation with different mineral premixes on blood parameter ^1^.

Items	Dietary Treatment ^2^			SEM ^3^	*p*-Value	
	Basal	Inorganic	Organic		Basal vs. Mineral	Inorganic vs. Organic
White blood cell, K/μL	4.00	6.82	9.92	2.071	0.32	0.28
Heterophil, K/μL	0.39	1.20	1.40	0.533	0.18	0.90
Lymphocyte, K/μL	3.24	4.70	7.49	1.311	0.25	0.77
Heterophil:Lymphocyte ratio	0.13	0.28	0.19	0.048	0.32	0.25
Red blood cell, M/μL	2.80	2.75	2.86	0.154	0.12	0.32
Hemoglobin, g/dL	8.97	8.68	8.72	0.323	0.20	0.27
Hematocrit, %	27.51	27.34	28.20	1.562	0.39	0.38

^1^ Data are least squares means of 10 replicate per treatment. ^2^ Basal = basal diet (no inclusion mineral premix); Inorganic = 1.0 g/kg inorganic mineral premix; Organic = 1.8 g/kg organic mineral premix. ^3^ Standard error of means.

**Table 5:** Effect of dietary supplementation with different mineral premixes on mineral concentrations in spleen, liver, eggshell, and whole blood ^1^.

Items		Dietary Treatment ^2^			SEM ^3^	*p*-Value	
		Basal	Inorganic	Organic		Basal vs. Mineral	Inorganic vs. Organic
		g/mg					
Liver	Iron	301.7 ^b^	513.1 ^ab^	645.5 ^a^	95.89	0.04	0.35
	Copper	5.94 ^b^	6.04 ^ab^	6.86 ^a^	1.481	0.78	0.04
	Zinc	95.2	152.6	150.5	22.03	0.11	0.95
	Manganese	8.5 ^b^	18.8 ^a^	19.4 ^a^	2.844	0.01	0.90
	Magnesium	543.3	708.5	677.8	73.49	0.12	0.77
Spleen	Iron	146.0 ^b^	255.2 ^a^	296.6 ^a^	24.38	<0.01	0.25
	Copper	2.73	3.33	4.10	0.481	0.12	0.28
	Zinc	58.4	98.5	100.4	17.26	0.08	0.94
	Manganese	1.01	1.86	1.66	0.511	0.25	0.79
	Magnesium	111.7 ^b^	177.0 ^ab^	227.5 ^a^	22.41	<0.01	0.14
Eggshell	Iron	10.3	11.1	11.3	1.107	0.53	0.92
	Copper	2.41 ^b^	2.53 ^a^	2.61 ^a^	0.050	0.02	0.18
	Zinc	4.22 ^b^	4.60 ^a^	4.84 ^a^	0.127	<0.01	0.29
	Manganese	2.38 ^b^	3.48 ^a^	3.63 ^a^	0.064	<0.01	0.32
	Magnesium	2.55 ^b^	2.85 ^ab^	3.00 ^a^	0.097	<0.01	0.21
Blood	Iron	492.7	510.2	509.2	26.95	0.42	0.35
	Copper	1.38	1.46	1.52	0.142	0.36	0.32
	Zinc	17.5	19.1	18.4	1.251	0.24	0.38
	Manganese	2.02	2.70	3.10	0.465	0.28	0.58
	Magnesium	125.8	135.4	145.1	4.431	0.35	0.27

^a,b^ Values in the same row with different superscripts differ significantly (*p* < 0.05). *n* = 10. ^1^ Data are presented as least squares means of 10 replicates per treatment. ^2^ Basal = basal diet (no inclusion mineral premix); inorganic = 1.0 g/kg inorganic mineral premix; organic = 1.8 g/kg organic mineral premix. ^3^ Standard error of means.

## Data Availability

Not applicable.
